# Education Research Training for Academic Emergency Medicine Educators

**DOI:** 10.5811/westjem.2021.9.54152

**Published:** 2021-12-17

**Authors:** Ryanne J. Mayersak, Lalena M. Yarris

**Affiliations:** Oregon Health & Science University, Department of Emergency Medicine, Portland, Oregon

## INTRODUCTION

This special issue of the *Western Journal of Emergency Medicine*, co-sponsored by the Council of Emergency Medicine Residency Directors (CORD) and the Clerkship Directors in Emergency Medicine (CDEM), serves as a snapshot of the current state of emergency medicine (EM) education research and focuses on relevant topics published by a diverse group of education scholars. Our field has seen marked increases in scholarship, publication venues, funding, and training opportunities for EM education research over the past decade.^1^–^3^ However, a lack of expertise in education research is still one of the main perceived barriers to educators reaching their scholarship goals.^4^–^6^ Educators who are new to research may not be aware of avenues to access the training, collaboration, and mentorship they need to achieve their scholarship goals. These avenues are now myriad and include everything from do-it-yourself episodic training, either in the digital space or in person, to longitudinal doctorate degree programs. Our aim in this piece is to describe available options for faculty development in education research, presented in the below table, along with references for exemplar programs. This table may be used by educators, mentors, and department leaders to determine the best fit for individual faculty development needs.

## Figures and Tables

**Table 1:** Opportunities for faculty development in education research.

Type	Description	Unique considerations (benefits/drawbacks)	Examples
Podcasts	Audio recordings of conversations or presentations about education research	Benefits those who prefer audio media, allows flexibility, may be accessed while doing other activities, allows the listener to feel connected to experts in the field and builds a sense of community.	KeyLIME podcasts^7^ Hot Topics in Med Ed Podcasts^8^
Blogs	Web-based repository for resources and discourse about education research	Convenient and searchable, may be peer-reviewed or crowd-sourced. Media may include articles, podcasts, videos, infographics, and discussion forums. Quality and accuracy may be variable.	ICE blog^9^
Webinars	Broadcast or recorded web-based training sessions that may be stand-alone, or part of a series	Often can be watched asynchronously, which adds flexibility. Lower cost than conferences. Scalable for a large number of learners.	AMEE Webinars^10^ GWU Education Research Modules^11^ CAEP Education Scholarship Committee Workshops^12^
Digital conversations	Digital platforms (such as twitter, WhatsApp, Slack) allow organizations to host scheduled discussions centered on education research	Because conversation occurs in real time, digital platform chats can allow researchers access to expertise and promote networking and community.	Twitter #MedEdChat series^13^ Institutional educational community exchanges (Slack platform)
Episodic training	Intermittent sessions held at institutions, through national organizations, or at national education conferences	Flexible, and allows faculty to select sessions based on needs or interests. May be difficult to translate into action and lack benefits of longitudinal programming and developing a community.	Institutional Education Grand Rounds SAEM Education Summit^14^ CAEP Education Academic Symposia^15^ McMaster Health Professions Education Research (HPER) course^16^ AAMC meeting and regional GEA workshops^17^,^18^
Certificate-type programs	Longitudinal programs, which may vary in duration and format, but provide an education scholarship curriculum	May result in more holistic training than episodic sessions, with increased ability to network and collaborate. Virtual or hybrid options may increase flexibility and decrease cost.	Institution-specific health professions education research certificate programs MERC, MERC at CORD^19^–^21^ ALiEM Faculty Incubator^22^,^23^ Harvard Macy Institute^24^ ARMED MedEd^25^
Post-graduate fellowships	Dedicated 1–2 year experience after residency training and prior to a faculty position	Fellowships vary in structure, duration, and focus. Dedicated 2-year fellowships may include an advanced degree. Opportunities for experiential learning and close mentorship.	EM education research fellowships^26^,^27^ Royal College of Physicians and Surgeons of Canada Area of Focused Competency (AFC) Clinician Educator Diploma Program^28^
Master’s Degree Programs	Dedicated master’s program that can be pursued at any point in career and confers a degree that is recognized externally	Options include MSEd, MHPE, MEHP, MCR, MMEd, and other master’s degree options. Programs vary in duration, format, and flexibility, with increasing virtual and hybrid options. Benefits include cohort and mentorship.	FAIMER database for master’s degree programs^29^
Doctorate Degree Programs	Dedicated doctorate program that can be pursued at any point in career and confers a degree that is recognized externally	Options include EdD, PhD, or other advanced graduate degrees. Highest level of training available. Programs vary in duration, format, and flexibility, some with virtual or hybrid options. Optimal for developing a program of research and mentorship.	FAIMER database for doctorate degree programs^30^

*KeyLIME*, Key Literature in Medical Education; *ICE*, International Clinical Educators; *AMEE*, Association for Medical Education in Europe; *GWU*, George Washington University

*SAEM*, Society for Academic Emergency Medicine; *MERC*, Medical Education Research Certification; *CORD*, Council of Residency Directors; *ARMED MedED*, Advanced Research Methodology Evaluation and Design in Medical Education; *EM*, emergency medicine; *FAIMER*, Foundation for Advancement of International Medical Education and Research
